# 矩阵热转移技术鉴定有机酸类代谢物的结合蛋白质

**DOI:** 10.3724/SP.J.1123.2023.07002

**Published:** 2024-07-08

**Authors:** Kejia LI, Yuying YE, Xiaolei ZHANG, Jiahua ZHOU, Yanan LI, Mingliang YE

**Affiliations:** 1.中国科学院大连化学物理研究所,中国科学院分离分析化学重点实验室, 辽宁 大连 116023; 1. CAS Key Laboratory of Separation Science for Analytical Chemistry, Dalian Institute of Chemical Physics, Chinese Academy of Science, Dalian 116023, China; 2.中国科学院大学,北京 100049; 2. University of Chinese Academy of Sciences, Beijing 100049, China

**Keywords:** 矩阵热转移技术, 有机酸类代谢物, 琥珀酸, 富马酸, 乳酸, 结合蛋白质, matrix thermal shift assay (mTSA), organic acid metabolites, succinate, fumarate, lactate, binding proteins

## Abstract

有机酸类代谢物在表观遗传、肿瘤发生和发展以及细胞内信号转导等方面发挥着重要作用,对这类代谢物在体内的结合蛋白质进行鉴定,将有助于从分子学层面理解和揭示它们的功能。本研究采用矩阵热转移技术(mTSA),在HeLa细胞裂解液中系统鉴定了3种有机酸类代谢物(琥珀酸、富马酸和乳酸)的结合蛋白质。首先,向细胞裂解液中加入一系列不同浓度的琥珀酸、富马酸或乳酸,在52 ℃下加热3 min,离心并收集上清蛋白质,进行酶解及后续质谱分析。在数据非依赖性采集(DIA)模式下,本研究在琥珀酸、富马酸和乳酸的mTSA实验中分别鉴定到5870、5744和5816个蛋白质;通过对蛋白质热稳定性的显著性差异值(*p*)和皮尔森相关系数平方值(*R*^2^)进行考察,该研究鉴定到了多个高可信度的有机酸类代谢物结合蛋白质。此外本研究发现,虽然富马酸和琥珀酸均能够与*α*-酮戊二酸依赖的双加氧酶(FTO)结合,但琥珀酸是FTO更强的竞争性抑制剂。除此之外,本研究还鉴定到了两个先前未曾报道过的乳酸结合蛋白质,即鸟氨酸转氨酶(OAT)和3-巯基丙酮酸硫转移酶(MPST),并通过溶剂诱导沉淀技术(SIP)和两种数据检验方法证明了其可信度;同时,OAT和MPST的发现有助于揭示乳酸在氨基酸合成和细胞内氧化还原平衡调节方面的重要作用。

## Abstract (en)

Organic acid metabolites exhibit acidic properties. These metabolites serve as intermediates in major carbon metabolic pathways and are involved in several biochemical pathways, including the tricarboxylic acid (TCA) cycle and glycolysis. They also regulate cellular activity and play crucial roles in epigenetics, tumorigenesis, and cellular signal transduction. Knowledge of the binding proteins of organic acid metabolites is crucial for understanding their biological functions. However, identifying the binding proteins of these metabolites has long been a challenging task owing to the transient and weak nature of their interactions. Moreover, traditional methods are unsuitable for the structural modification of the ligands of organic acid metabolites because these metabolites have simple and similar structures. Even minor structural modifications can significantly affect protein interactions. Thermal proteome profiling (TPP) provides a promising avenue for identifying binding proteins without the need for structural modifications. This approach has been successfully applied to the identification of the binding proteins of several metabolites. In this study, we investigated the binding proteins of two TCA cycle intermediates, i.e., succinate and fumarate, and lactate, an end-product of glycolysis, using the matrix thermal shift assay (mTSA) technique. This technique involves combining single-temperature (52 ℃) TPP and dose-response curve analysis to identify ligand-binding proteins with high levels of confidence and determine the binding affinity between ligands and proteins. To this end, HeLa cells were lysed, followed by protein desalting to remove endogenous metabolites from the cell lysates. The desalted cell lysates were treated with fumarate or succinate at final concentrations of 0.004, 0.04, 0.4, and 2 mmol/L in the experimental groups or 2 mmol/L sodium chloride in the control group. Considering that the cellular concentration of lactate can be as high as 2-30 mmol/L, we then applied lactate at final concentrations of 0.2, 1, 5, 10, and 25 mmol/L in the experimental groups or 25 mmol/L sodium chloride in the control group. Using high-sensitivity mass spectrometry coupled with data-independent acquisition (DIA) quantification, we quantified 5870, 5744, and 5816 proteins in succinate, fumarate, and lactate mTSA experiments, respectively. By setting stringent cut-off values (i.e., significance of changes in protein thermal stability (*p*-value)<0.001 and quality of the dose-response curve fitting (square of Pearson’s correlation coefficient, *R*^2^)>0.95), multiple binding proteins for these organic acid metabolites from background proteins were confidently determined. Several known binding proteins were identified, notably fumarate hydratase (FH) as a binding protein for fumarate, and *α*-ketoglutarate-dependent dioxygenase (FTO) as a binding protein for both fumarate and succinate. Additionally, the affinity data for the interactions between these metabolites and their binding proteins were obtained, which closely matched those reported in the literature. Interestingly, ornithine aminotransferase (OAT), which is involved in amino acid biosynthesis, and 3-mercaptopyruvate sulfurtransferase (MPST), which acts as an antioxidant in cells, were identified as lactate-binding proteins. Subsequently, an orthogonal assay technique developed in our laboratory, the solvent-induced precipitation (SIP) technique, was used to validate the mTSA results. SIP identified OAT as the top target candidate, validating the mTSA-based finding that OAT is a novel lactate-binding protein. Although MPST was not identified as a lactate-binding protein by SIP, statistical analysis of MPST in the mTSA experiments with 10 or 25 mmol/L lactate revealed that MPST is a lactate-binding protein with a high level of confidence. Peptide-level empirical Bayes *t*-tests combined with Fisher’s exact test also supported the conclusion that MPST is a lactate-binding protein. Lactate is structurally similar to pyruvate, the known binding protein of MPST. Therefore, assuming that lactate could potentially occupy the binding site of pyruvate on MPST. Overall, the novel binding proteins identified for lactate suggest their potential involvement in amino acid synthesis and redox balance regulation.

有机酸类代谢物是一类具有酸性性质的代谢物,常见的有机酸类代谢物包括乳酸、丙酮酸、酮戊二酸和富马酸等。越来越多的研究表明,这类代谢物不仅是细胞能量代谢的产物,而且它们在表观遗传^[1]^、肿瘤的发生和发展^[2]^以及细胞内信号转导^[3]^等方面都发挥着重要的作用。蛋白质是生命活动的主要承担者,许多有机酸类代谢物都需要通过与蛋白质结合来调控生命活动^[2,4]^。因此,对有机酸类代谢物的结合蛋白质进行鉴定,将有助于深入理解这类代谢物的作用机制,并发现新的功能。

目前,小分子(包括代谢物)结合蛋白质的鉴定方法主要有两种,分别是小分子结构修饰方法和小分子结构免修饰方法。小分子结构修饰方法主要是通过在小分子上修饰一个可被固定或富集的标签,从而富集出与小分子结合的蛋白质,该方法也被称为亲和富集法^[5]^。在去除非特异性结合蛋白质的过程中,小分子结构修饰方法需要较强的洗涤条件,而一些与蛋白质相互作用较弱的小分子难以通过该方法进行鉴定。为了解决这个问题,Parker等^[6]^在修饰后的小分子上进一步引入了光反应基团,将非共价相互作用转变为强共价相互作用,从而有利于弱结合蛋白质的鉴定。然而,对于结构简单且非常相似的有机酸类代谢物,任何结构上的改变都有可能导致其结合蛋白质发生巨大变化,因此小分子结构修饰方法难以发挥作用。近些年发展的小分子结构免修饰方法,如蛋白质氧化速率稳定性技术(stability of proteins from rates of oxidation, SPROX)^[7]^、细胞内热转移技术(cellular thermal shift assay, CETSA)^[8]^、热蛋白质组技术(thermal proteome profiling, TPP)^[9]^以及限制性酶切联合质谱技术(limited proteolysis coupled to mass spectrometry, LiP-MS)^[10]^等,受到了国内外同行的广泛关注,尤其是TPP在药物结合蛋白质鉴定^[11]^、代谢物结合蛋白质鉴定^[12]^以及功能性翻译后修饰筛选^[13,14]^等方面得到了广泛应用。我们课题组^[15]^在TPP的基础上发展了单温度点TPP与多个小分子剂量处理结合的矩阵热转移技术(matrix thermal shift assay, mTSA),仅采用一个温度点(52 ℃)使蛋白质变性,并利用非标记的数据非依赖型采集(data-independent acquisition, DIA)模式对小分子的结合蛋白质进行鉴定。该技术大幅提高了传统TPP方法的通量,同时也提高了结合蛋白质的鉴定灵敏度;此外,该技术采用多个小分子处理剂量结合剂量响应曲线分析,不仅能够进一步提高结合蛋白质鉴定的可信度,还能用于计算小分子与结合蛋白质之间的亲和力。

琥珀酸和富马酸均为三羧酸(TCA)循环的中间产物,并且琥珀酸是富马酸的上游底物,琥珀酸经琥珀酸脱氢酶脱氢后即可形成富马酸,因此二者的结构非常相似。不同于琥珀酸和富马酸的二羧酸结构,乳酸是糖酵解过程中产生的、仅含有3个碳原子的一元酸。上述3种有机酸类代谢物可用于化学修饰的空间都非常小,因此难以通过小分子结构修饰方法来鉴定其结合蛋白质。

本研究采用mTSA技术,在HeLa细胞裂解液中系统鉴定了3种有机酸类代谢物(琥珀酸、富马酸和乳酸)的结合蛋白质,并分别测定了3种有机酸类代谢物与其结合蛋白质之间的亲和力。对于新鉴定到的未知结合蛋白质,使用本课题组^[16]^发展的mTSA正交技术(即溶剂诱导沉淀技术(solvent-induced precipitation, SIP))和两种数据检验方法进行验证。本研究为琥珀酸、富马酸和乳酸在生命活动中的功能研究提供了有价值的线索。

## 1 实验部分

### 1.1 仪器、试剂与材料

配有Dionex UltiMate 3000 RSLC微升液相色谱系统的Q-Exactive Exploris 480质谱仪、NanoDrop超微量分光光度计、Steri-Cycle CO_2_ Incubator 371细胞培养箱、Heraeus Multfugei X1R高速离心机、Multi-Therm H5000-HC振荡器、SPD SpeedVAC SPD131DDA真空冻干浓缩仪(美国Thermo Fisher Scientific公司); BioTek酶标仪(美国Agilent公司);聚合酶链式反应(PCR)热循环仪(美国Bio Rad公司)。

琥珀酸、L型乳酸(以下均简称为乳酸)、盐酸胍(GdmCl)、三(2-羧乙基)膦盐酸盐(TCEP)、2-氯乙酰胺(CAA)、氯化钠(NaCl)、碳酸氢铵(NH_4_HCO_3_)、4-羟乙基哌嗪乙磺酸(HEPES)、蛋白酶抑制剂Cocktail、三氟乙酸(TFA)、甲酸(FA)(纯度均大于98%,美国Sigma-Aldrich公司);富马酸(纯度大于98%)(上海生工生物股份有限公司);丙酮、乙酸(分析纯,大连博诺生物化学试剂厂);无水乙醇(分析纯,天津富宇精细化工有限公司);磷酸盐缓冲液(PBS)粉末(北京索莱宝科技有限公司);乙腈(ACN)、甲醇(MeOH)、超纯水(质谱级,德国Merck公司);胰蛋白酶(trypsin,测序级,美国Promega公司);二喹啉甲酸(BCA)法试剂盒、青霉素-链霉素储存液(上海碧云天生物技术有限公司); iRT(indexed retention time)标准肽段混合物(瑞士Biognosys公司); DMEM培养基、牛血清(BS)(美国Gibco公司);细胞培养皿(美国Corning公司); Zeba^TM^ Spin Desalting Plates蛋白质除盐柱(7K MWCO,美国Thermo Fisher Scientific公司); 10 kDa超滤离心管(德国Sartorius公司)。除液相色谱-串联质谱使用的流动相溶液外,其他溶液配制所用的超纯水均由Milli-Q超纯水机(美国Millipore公司)制备。

变性剂溶液的配制:准确称取38.216 g GdmCl和0.714 g HEPES,用超纯水溶解,并定容至50 mL。

### 1.2 细胞培养和蛋白质提取

将HeLa细胞复苏于含有10% BS、100 U/mL青霉素和100 U/mL链霉素的DMEM完全培养基中,之后在37 ℃、5% CO_2_、湿度适宜的细胞培养箱中进行贴壁培养;待细胞铺满培养皿面积的80%后,离心去除培养基,并用预冷的PBS清洗细胞,重复清洗3次;在冰沙上将细胞轻轻刮取至15 mL离心管中,之后在1000 g下离心5 min,取下层沉淀细胞,并于-80 ℃冰箱中保存备用。

将HeLa细胞重悬于裂解缓冲液(含有1%蛋白酶抑制剂Cocktail的PBS)中,并吹散;使用液氮反复冻融法提取细胞中的蛋白质,具体操作如下:将细胞与裂解缓冲液的混合悬浮液投入至液氮中,快速冷冻2 min,随后立即转移至37 ℃水浴中,融化2 min,直至样品中约60%的内容物融化,再将样品放至冰沙上至完全融化;重复上述过程3次后,将HeLa细胞在20000 g、4 ℃下离心10 min,小心移取上清液至新的离心管中,所得溶液即为保持了蛋白质原有构象的HeLa细胞裂解液。

### 1.3 内源性代谢物的去除

考虑到细胞内原有的高浓度代谢物可能会影响结合蛋白质的鉴定以及亲和力的测定,因此本实验采用蛋白质除盐柱对HeLa细胞裂解液进行除盐操作,具体实验步骤如下:取蛋白质除盐柱,在1000 g下离心2 min以去除其中的储存液,加入1 mL裂解缓冲液润洗蛋白质除盐柱,之后在1000 g下离心2 min,重复此步骤3次;移取700 μL HeLa细胞裂解液至蛋白质除盐柱中,在1000 g下离心2 min,所得到的过滤液即为去除了内源性代谢物的细胞裂解液;随后使用BCA法测定上述细胞裂解液中的蛋白质含量,并用裂解缓冲液将其质量浓度调整为4.5 mg/mL。

### 1.4 mTSA分析

将上述HeLa细胞裂解液分别与一系列不同浓度的有机酸类代谢物分子进行混合,并在室温下孵育50 min。对于琥珀酸和富马酸,实验组中二者的终浓度分别为0.004、0.04、0.4、2 mmol/L;在对照组中加入20 mmol/L的氯化钠水溶液,使氯化钠的终浓度为2 mmol/L。对于乳酸,实验组中其终浓度分别为0.2、1、5、10、25 mmol/L;在对照组中加入100 mmol/L的氯化钠水溶液,使氯化钠的终浓度为25 mmol/L。孵育完毕后,将所有样品均分为4份,在52 ℃下加热3 min,然后在室温下冷却2 min;之后将所有样品在20000 g、4 ℃下离心10 min,并收集上清蛋白质样品至离心管中。

在收集到的蛋白质样品中分别加入3倍体积的变性剂溶液(pH 8.0),使蛋白质变性。向变性后的蛋白质溶液中分别加入终浓度为10 mmol/L的TCEP和40 mmol/L的CAA,并在95 ℃下加热5 min,进行还原烷基化。利用基于滤膜辅助的样品制备流程(filter aided sample preparation protocol, FASP)^[17]^对所得蛋白质溶液进行处理,具体操作步骤如下:(1)将蛋白质溶液转移至10 kDa 500 μL的超滤管中,在14000 g、25 ℃下离心40 min,并尽量去除超滤离心管中的溶液;(2)向超滤管中加入200 μL 10 mmol/L的NH_4_HCO_3_溶液,在14000 g、25 ℃下离心30 min,重复两次,随后更换新的滤液收集管,并向超滤管中加入80 μL 10 mmol/L的NH_4_HCO_3_溶液;(3)以1∶30(胰蛋白酶∶蛋白质)的质量比加入胰蛋白酶,在37 ℃下酶解过夜,之后在14000 g下离心30 min,收集酶解肽段,并冻干保存。

### 1.5 SIP分析

将去除了内源性代谢物的HeLa细胞裂解液分为两组,分别与终浓度为25 mmol/L的乳酸(实验组)和终浓度为25 mmol/L的氯化钠水溶液(对照组)混合,在室温下孵育50 min。孵育完毕后,将所有样品均分为4份,并向每个样品中加入一定体积的沉淀液(丙酮-乙醇-乙酸(500∶500∶1, v/v/v)),使沉淀液的最终体积分数为9%。之后将所有样品在20000 g、4 ℃下离心10 min,收集上清蛋白质样品至离心管中。随后的蛋白质变性、还原烷基化以及酶解等实验步骤同1.4节。

### 1.6 液相色谱-串联质谱分析

将酶解后的肽段样品复溶在0.1%甲酸水溶液中,以9∶1的体积比加入iRT标准肽段混合物,并利用Nanodrop超微量分光光度计测定肽段的浓度。将10 μg肽段样品上样至ACQUITY UPLC Peptide CSH C18商品化色谱柱(150 mm×1 mm, 1.7 μm)中,流动相A为0.1%甲酸水溶液,流动相B为乙腈-甲酸-水(80∶0.1∶20, v/v/v),柱温为55 ℃。梯度洗脱程序:0~0.5 min, 4%B, 75 μL/min; 0.5~1.0 min, 4%B~6%B, 50 μL/min; 1.0~80.0 min, 6%B~32%B, 50 μL/min; 80.0~93.5 min, 32%B~45%B, 50 μL/min; 93.5~94.0 min, 45%B~90%B, 50 μL/min; 94.0~98.0 min, 90%B, 50 μL/min; 98.0~100.0 min, 4%B, 75 μL/min。液相色谱与质谱之间装有高场非对称波形离子迁移(FAIMS)装置,以去除一价离子,FAIMS装置的补偿电压为-45 V,总载气流量为3.5 L/min。

采用正离子扫描模式和DIA采集模式,一级质谱扫描范围为*m/z* 350~1400,共有24个DIA分段窗口被依次扫描(*m/z* 400~1000),一级质谱分辨率设置为120000 (*m/z* 200),离子最大注入时间为45 ms,允许注入最大电荷数为3×10^6^。归一化碰撞碎裂能量(NCE)设置为30%,二级质谱分辨率设置为30000(*m/z* 200),离子最大注入时间设置为“auto”,允许注入最大电荷数为2×10^6^。

### 1.7 数据库检索

将得到的质谱文件加载到Spectronaut软件(版本17)中,使用不依赖于谱图库的检索模式(即directDIA)进行检索。所使用的数据库为人源蛋白质数据库(包含20168个条目,www.uniprot.org)。所使用的酶设置为“Trypsin”,最大漏切数设置为2,固定修饰设定为半胱氨酸(C)上的氨基甲酰甲基化(carbamidomethyl),可变修饰设置为甲硫氨酸(M)的氧化以及蛋白质N端的乙酰化,其他参数保持为Spectronaut软件的默认参数,缺失值的插入方式为“Global Imputing”;前体离子和蛋白质鉴定的假阳性率(false discovery rate, FDR)均设置为1%;在排除掉干扰肽段后,选择丰度最高的3条肽段进行蛋白质的定量,使用“Local normalization”策略对蛋白质定量结果进行归一化处理。

## 2 结果与讨论

### 2.1 mTSA蛋白质组鉴定深度及定量重复性评估

琥珀酸在细胞中的生理浓度为0.5~1 mmol/L^[18]^,为模拟代谢异常情况下的琥珀酸浓度,本实验将琥珀酸最大处理浓度设置为2 mmol/L。富马酸在细胞中的生理浓度约为0.1 mmol/L^[18]^,据文献[19]报道,富马酸和琥珀酸均能竞争性地与依赖于*α*-酮戊二酸的双加氧酶结合,为平行比较富马酸和琥珀酸的结合蛋白质及结合亲和力的差异,在富马酸的mTSA实验中采用与琥珀酸相同的浓度处理梯度。乳酸在细胞中的生理浓度为2~30 mmol/L^[20⇓-22]^,因此在乳酸的mTSA实验中,将乳酸的终浓度设置为0.2、1、5、10、25 mmol/L。

如图1所示,在基于DIA的定量模式下,分别在琥珀酸、富马酸和乳酸的mTSA实验中鉴定到了5809、5744和5816个蛋白质,表明本实验的mTSA蛋白质数据集具有较深的蛋白质组覆盖度;此外,每组实验中蛋白质定量结果之间的皮尔森相关系数平方值(square of Pearson’s correlation coefficient, *R*^2^)均大于0.95,表明实验操作的平行性及DIA定量结果的重复性均较好。

**图1:**
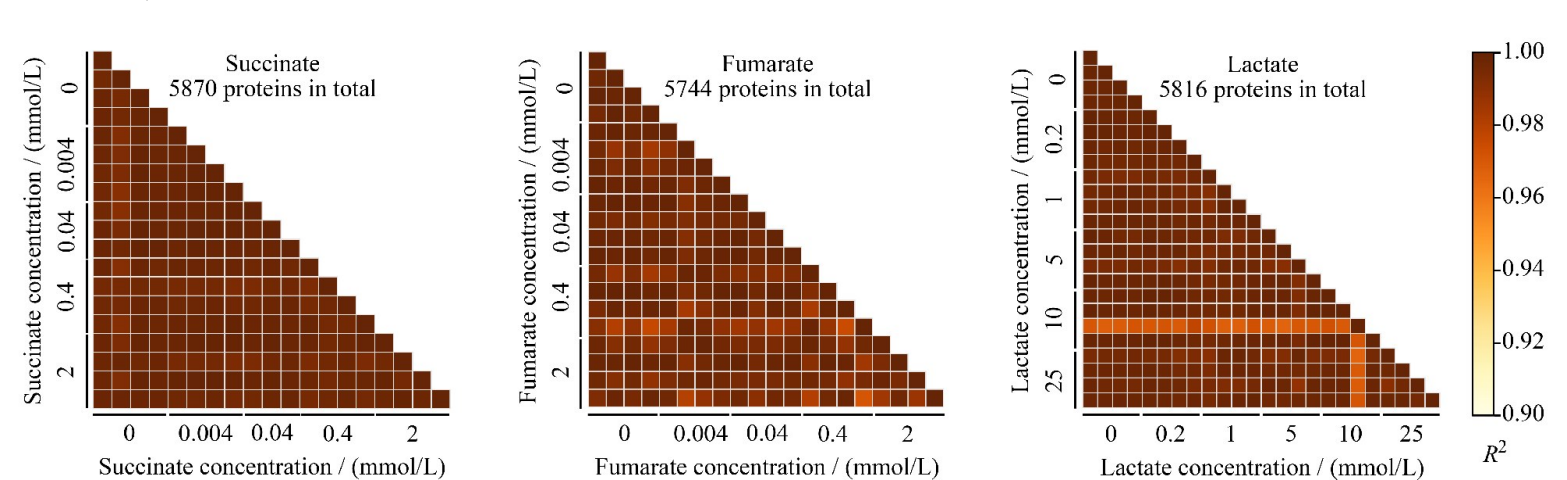
mTSA蛋白质数据集对琥珀酸、富马酸和乳酸的鉴定深度及定量重复性评估

### 2.2 已知结合蛋白质的鉴定及亲和力的计算

为了鉴定琥珀酸、富马酸和乳酸的所有潜在结合蛋白质,首先对最高处理浓度下变化显著的蛋白质进行分析。如图2a所示,在2 mmol/L琥珀酸处理浓度下,利用经验贝叶斯显著性检验(empirical Bayes *t*-test)来筛选显著性差异值(*p*-value, *p*)<0.001的蛋白质,结果共鉴定到了琥珀酸的2个潜在结合蛋白质;同理,按照上述筛选标准,在富马酸和乳酸的最高处理浓度下,分别鉴定到了富马酸的11个潜在结合蛋白质(图2b)和乳酸的5个潜在结合蛋白质(图2c)。然而,当使用其他多个处理浓度下的数据来分析上述潜在结合蛋白质时,部分潜在结合蛋白质随处理浓度的增加,其稳定性并没有呈现出规律性的增加,表明上述潜在结合蛋白质在最高处理浓度下的变化可能是随机的。因此,仅凭最大处理浓度下的筛选标准(*p*<0.001),并不能筛选出完全可信的结合蛋白质。

**图2:**
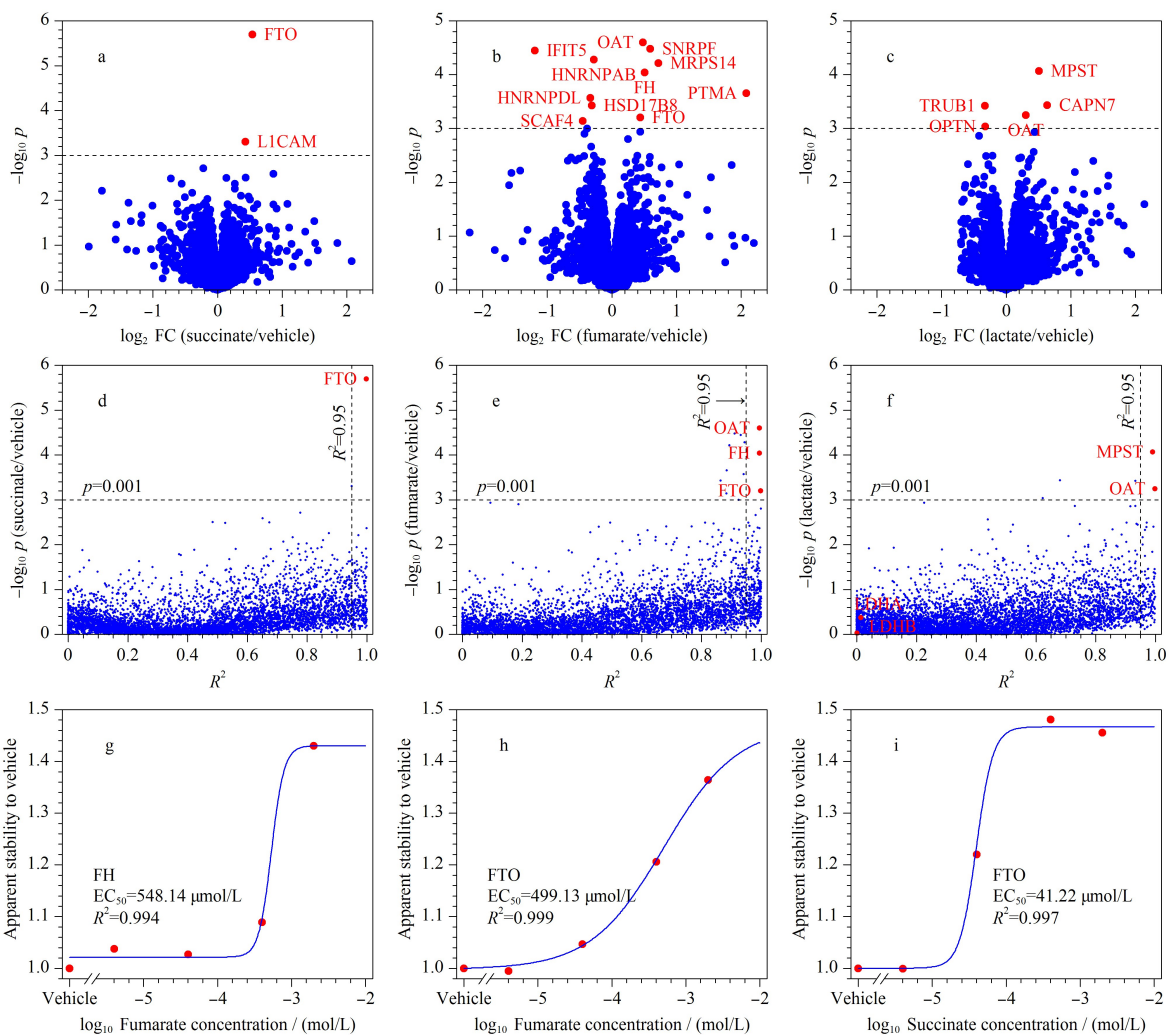
琥珀酸、富马酸和乳酸已知结合蛋白质的鉴定及亲和力的计算

为了筛选出更为可信的结合蛋白质,沿用mTSA^[15]^方法,绘制所有蛋白质的剂量响应曲线,其中*R*^2^代表拟合质量,并以*R*^2^>0.95和*p*<0.001作为筛选条件来确定最终的结合蛋白质。结果如图2d和2e所示,在琥珀酸和富马酸的mTSA实验中分别筛选到了1个和3个潜在结合蛋白质;并且,其中大部分的潜在结合蛋白质已被文献[23⇓-25]报道,能够与琥珀酸或富马酸结合。

在TCA循环中,富马酸脱氢酶(FH)能够催化富马酸水合形成苹果酸,据文献[23]报道,FH与富马酸之间的亲和力(以半数有效配体浓度(EC_50_)作为计算依据)为320~420 μmol/L。本实验对富马酸浓度及蛋白质在每个富马酸处理浓度下热稳定性变化的幅度进行拟合,以此来计算FH与富马酸之间的亲和力,结果如图2g所示。通过计算所得到的亲和力为548.14 μmol/L,与文献[23]报道结果接近,表明了实验结果的可靠性。FTO是以*α*-酮戊二酸为底物的双加氧酶,在加入琥珀酸或富马酸后,FTO的热稳定性显著增加(*p*<0.001且log_2_ FC>0),表明琥珀酸和富马酸均能够与FTO结合(图2d和2e),该结果与文献[19]报道的结论一致,即琥珀酸和富马酸均可以作为*α*-酮戊二酸的竞争型抑制剂。如图2h和2i所示,FTO与富马酸和琥珀酸之间的亲和力分别为499.13 μmol/L和41.22 μmol/L,表明琥珀酸是FTO更强的竞争型抑制剂。此外,实验还发现富马酸可以与鸟氨酸转氨酶(OAT)结合(图2e),虽然OAT不是双加氧酶,但OAT能够将*α*-酮戊二酸转化为谷氨酸^[26]^,从而参与氨基酸合成,表明富马酸也可以与其他以*α*-酮戊二酸为底物的酶或蛋白质结合。

对于乳酸,虽然本实验鉴定到了已知的乳酸结合蛋白质,即乳酸脱氢酶A (LDHA)和乳酸脱氢酶B (LDHB),但在本实验中,LDHA和LDHB并没有产生热稳定性变化(图2f)。在与乳酸结合之前,乳酸脱氢酶需先与还原型烟酰胺腺嘌呤二核苷酸(NADH)结合形成复合物^[27]^,而实验所用到的细胞裂解液已去除了内源性代谢物(如NADH等),这可能会导致乳酸脱氢酶无法与乳酸结合。因此,在本实验中无法将LDHA和LDHB鉴定为乳酸的潜在结合蛋白质。此外,本工作鉴定到了两个先前未曾报道过的乳酸潜在结合蛋白质,即OAT和3-巯基丙酮酸硫转移酶(MPST)(图2f)。

### 2.3 未知乳酸结合蛋白质的验证

#### 2.3.1 SIP验证

SIP是一种与mTSA正交的配体结合蛋白质鉴定技术,其原理是当配体(如有机酸类代谢物)与蛋白质结合后,会使蛋白质对有机溶剂的耐受能力增强。为了进一步验证OAT和MPST是乳酸的结合蛋白质,首先采用SIP方法进行验证^[16]^。在实验组和对照组中分别加入相同体积的沉淀液,与乳酸结合后的蛋白质由于稳定性增加而更不容易沉淀,留在上清液中,最终通过比较实验组和对照组上清液中的蛋白质丰度来确定乳酸的结合蛋白质。

如图3a所示,在乳酸和氯化钠处理组中,各样品间蛋白质定量结果的*R*^2^均大于0.96,表明SIP实验操作的平行性及DIA定量结果的重复性较好。经过经验贝叶斯显著性检验发现,在加入25 mmol/L乳酸后,OAT的热稳定性变化最为显著(-log_10_ *p*=6.95, log_2_ FC=1.07)(图3b),即在SIP实验中,OAT被进一步鉴定为乳酸的结合蛋白质,并成功验证了乳酸的mTSA实验结果。此外,MPST在SIP实验中没有发生显著性变化(-log_10_ *p*=0.906, log_2_ FC=0.148)(图3b),可能是因为SIP和mTSA实验在结合蛋白质的鉴定方面具有一定的互补性^[28]^。

**图3:**
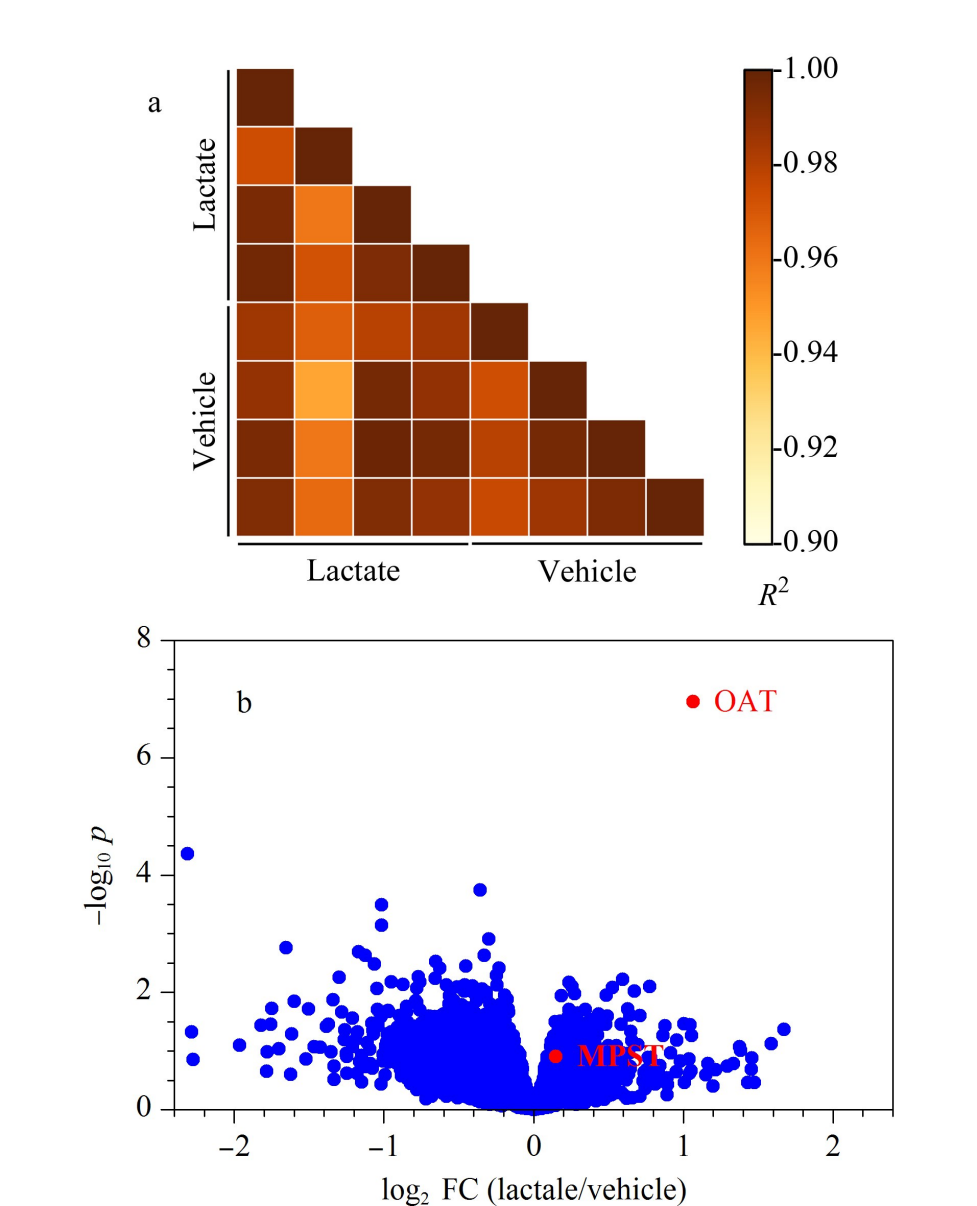
未知乳酸结合蛋白质的验证

#### 2.3.2 MPST的多重数据检验

为了证明MPST为乳酸的结合蛋白质,进一步从数据检验的角度对mTSA实验中MPST的变化可信度进行分析。首先在蛋白质层次上进行数据检验和分析,如前所述,MPST是通过25 mmol/L乳酸处理浓度下的经验贝叶斯显著性检验*p*值以及剂量响应曲线*R*^2^共同筛选出来的,因此后续进一步对MPST在10 mmol/L乳酸处理浓度下的经验贝叶斯显著性检验结果进行考察。如图4a所示,在10 mmol/L乳酸处理浓度下,MPST同样被鉴定为发生了显著变化的蛋白质(按照*p*从大至小排序,MPST排在第二位)。从统计学角度来讲,在25 mmol/L乳酸处理浓度下,随机挑中显著性变化排名前二的蛋白质的概率为2/5791(图4b);在10 mmol/L乳酸处理浓度下,随机挑中显著性变化排名前二的蛋白质的概率同样也为2/5791,则在两次独立挑选事件中,选中同一个蛋白质的概率为(2/5791)^2^,即该事件随机发生的概率为1.12×10^-7^。因此,上述实验结果可证明MPST是高可信度的乳酸结合蛋白质。

**图4:**
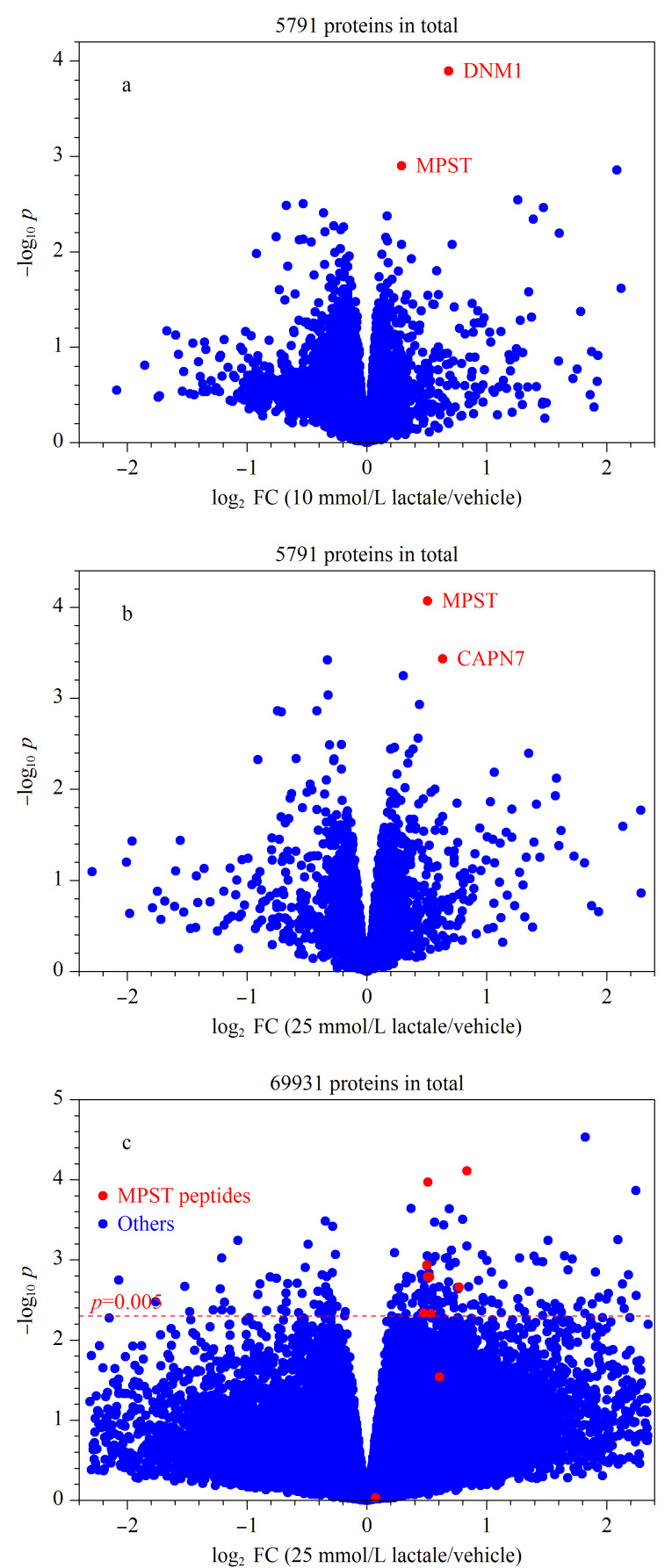
MPST的多重数据检验

之后在肽段层次上对MPST的可信度进行检验。如图4c所示,若将*p*<0.005的肽段定义为显著性变化的肽段,则利用mTSA实验共鉴定到的10个MPST肽段中,有8条肽段为显著性变化肽段(占比为80%);同时,在mTSA实验中共鉴定到了69931个肽段,其中仅有207个肽段为显著性变化肽段(*p*<0.005,占比为0.3%),结果表明显著性变化肽段在MPST中产生了明显富集。进一步采用费歇尔精确检验(Fisher’s exact test)对显著性变化肽段在MPST中的分布显著性进行分析,在鉴定到的69931个肽段中随机取10个肽段,共有7.70×10^41^种选择;而在鉴定到的69931个肽段中随机取10个肽段,其中8个肽段满足*p*<0.005、2个肽段满足*p*>0.005,则共有1.77×10^23^种选择。由此可知,在MPST的10个肽段中,若有8个肽段满足*p*<0.005且2个肽段满足*p*>0.005,则该事件随机发生的概率为2.30×10^-19^,这一极小概率事件证明了MPST是高可信度的乳酸结合蛋白质。MPST能够将2-酮-3-巯基丙酸转化为丙酮酸,从而发挥抗氧化作用^[29]^;而乳酸与丙酮酸在结构上具有相似性,由此推断,乳酸极有可能会竞争性地占据丙酮酸与MPST的结合位点,从而实现对MPST的功能调控,起到调节细胞内氧化还原反应的作用。

## 3 结论

本研究利用mTSA技术成功鉴定到了多个高可信度的3种有机酸类代谢物(琥珀酸、富马酸和乳酸)结合蛋白质。除了鉴定到的已知结合蛋白质外,本研究还鉴定到了两个先前未曾报道过的乳酸结合蛋白质(OAT和MPST),并通过SIP和多重数据检验方法将二者鉴定为高可信的乳酸结合蛋白质,为后续的乳酸功能研究提供了重要线索。但需要注意的是,还有很多能够与富马酸、琥珀酸或乳酸结合的蛋白质在本工作中没有被鉴定为潜在结合蛋白质,一方面可能是有机酸类代谢物与其相应结合蛋白质的结合需要其他辅因子参与,另一方面可能是mTSA技术无法用于鉴定对热处理敏感的结合蛋白质,因此未来需要发展其他互补方法来更全面地探索有机酸类代谢物在细胞内的作用网络。
